# ﻿*Suillusbovinus* sesquiterpenes stimulate root growth and ramification of host and non-host plants by coordinating plant auxin signaling pathways

**DOI:** 10.3897/imafungus.16.142356

**Published:** 2025-03-24

**Authors:** Wanyan Feng, Xueguang Sun, Guiyun Yuan, Guijie Ding

**Affiliations:** 1 Institute for Forest Resources & Environment of Guizhou, Guizhou University, Guiyang 550025, China Guizhou University Guiyang China; 2 College of Forestry, Guizhou University, Guiyang 550025, China Guizhou University Guiyang China

**Keywords:** Auxin signaling, lateral root, presymbiosis, sesquiterpenes, volatile organic compounds

## Abstract

Prior to physical contact, ectomycorrhizal (ECM) fungi can regulate plant root growth and ramification by emitting volatile organic compounds (VOCs). However, the underlying mechanisms of these VOC effects, as well as the key signaling molecules within the VOC blends, are largely unknown. Under sterile conditions, we studied the effects of the *Suillusbovinus*VOCs on the root growth of *Pinusmassoniana* or *Arabidopsisthaliana* before physical contact. Exogenously added auxin inhibitors and auxin-related mutants were used to explore the role of auxin in the promotion of plant root development by *S.bovinus*VOCs. *S.bovinus*VOCs stimulated host *P.massoniana* and non-host *A.thaliana* lateral root formation (LRF). Although these effects were independent of the host, they exhibited a symbiotic fungal-specific feature. Sesquiterpenes (SQTs) were the main *S.bovinus* VOC component that promoted LRF in plants. Two SQTs, α-humulene and β-cedrene, utilized different auxin pathways to promote plant root growth but did not affect the formation of an ECM symbiotic relationship between *P.massoniana* and *S.bovinus*. These findings enhance our understanding of the role played by SQTs in the signal recognition mechanism during the ECM presymbiotic stage and their role in promoting plant growth.

## ﻿Introduction

Most plant root systems form a symbiotic relationship with mycorrhizal fungi, facilitating nutrient and water uptake, enhancing resilience to environmental stress, and promoting plant growth (Smith and Read 2008; Martin and van Der Heijden 2024). In forest ecosystems, ectomycorrhizal (ECM) symbiosis is a prevalent association, in which ECM fungi colonize tree roots and establish a Hartig net and mantle structure (van Der Heijden et al. 2015; Martin et al. 2016).

Efficient mutual recognition between host roots and ECM fungi via signaling molecules at the presymbiotic stage (before physical contact) is the prerequisite step for the formation of an ECM symbiosis (Garcia et al. 2015; Raudaskoski and Kothe 2015; Kothe and Turnau 2018). These signaling molecules can elicit responses, such as stimulating lateral root formation (LRF) (Felten et al. 2009; Ditengou et al. 2015), inducing spore germination (Kikuchi et al. 2007), and promoting directional growth (Horan and Chilvers 1990) to facilitate ECM mutualistic relationships. Volatile organic compounds (VOCs) are ideal signaling molecules owing to their low molecular weight (< 300 Da), low boiling point, strong lipophilicity, and diffusivity (Schmidt et al. 2015; Fincheira and Quiroz 2018). Research has shown that VOCs play a crucial role in the signal recognition process during the presymbiotic stage of ECM formation (Martin et al. 2001; Garcia et al. 2015; Pellegrin et al. 2019) and even promote ECM fungal colonization at the symbiotic stage (Plett et al. 2024). There is increasing evidence that ECM fungi can induce host plants and *Arabidopsisthaliana*, a non-host plant, to form lateral roots without making physical contact with the roots by releasing VOCs (Felten et al. 2009; Splivallo et al. 2009; Ditengou et al. 2015). This suggests that the effects of ECM fungal VOCs are not host-specific (Feng et al. 2022); however, whether the host plant’s response to VOCs is fungus–specific has yet to be determined.

Auxin is essential in regulating plant root development (Saini et al. 2013). Previous studies suggest that VOCs emitted by ECM fungi promote LRF via the auxin pathway (Ivanchenko et al. 2008; Stepanova et al. 2008; Splivallo et al. 2009; Sun et al. 2009). However, some research does not support this view. For example, sesquiterpene (SQT) (–)-thujopsene released by *Laccariabicolor* stimulates LRF independently of the auxin pathway (Ditengou et al. 2015). This discrepancy may arise from variations in the types of VOCs released by different microbes. Interestingly, SQTs produced by other symbiotic fungi (non-ECM fungi) have been shown to depend on the auxin pathway for the regulation of LRF (Pérez-Flores et al. 2017; Tyagi et al. 2019; Li et al. 2022; He et al. 2023), indicating that this difference is also related to the specific plant–microbe symbiotic association.

Although many studies have investigated the role of microbial VOCs in plant–microbe interactions, studies involving ECM fungi have mainly focused on a few common combinations (such as *Populus*–*L.bicolor*). This has greatly limited the development and use of volatile resources produced by ECM fungi, which lag behind our understanding of the ECM symbiotic mechanism. *Suillus* species form symbiotic relationships with members of the *Pinaceae* (Policelli et al. 2019; Perez-Pazos et al. 2021) and are essential for colonization, diffusion, and invasion by conifer species (Rudawska et al. 2018; Ning et al. 2021). Meanwhile, *Suillus* is also considered a model system for studying ECM associations (Lofgren et al. 2024). *Pinusmassoniana* is a vital timber and ecological tree species in China (Zhou 2001) and a typical ECM host. In a previous investigation, we observed that *Suillusbovinus* not only has ecological and economic value but also is one of the dominant edible mycorrhizal fungi in *P.massoniana* plantation (Sun et al. 2019). Previous studies have mostly focused on the ability of *S.bovinus* to improve the growth and stress adaptability of *P.massoniana* (Chen et al. 2022), but little is known about the mechanism of this symbiosis, especially the recognition mechanism of VOCs before physical contact has been made.

In this study, we sought to understand the interaction between *S.bovinus* and the roots of *P.massoniana* or *A.thaliana* through VOC prior to physical contact. Our aim was to identify the main VOCs and mechanisms involved in this interaction. We identified signaling molecules from VOC blends emitted by ECM fungus *S.bovinus* coordinate auxin signaling pathways to promote LRF in both host *P.massoniana* and nonhost *A.thaliana*. Two SQTs, α-humulene and β-cedrene, engage distinct auxin pathways to enhance plant root growth but do not affect the ECM symbiotic relationship between *P.massoniana* and *S.bovinus*. Our findings provide a theoretical basis for understanding signal recognition mechanisms during the presymbiotic stage of the ECM interaction.

## ﻿Materials and methods

### ﻿Plant and fungal materials

The ECM fungi *Suillusbovinus*, *Suillusluteus*, and *Sclerodermacitrinum*; the dark septate endophytes (DSE) *Phialocephalafortinii*, and the saprophytic fungus *Lycoperdonperlatum* were provided by the Microbiology Laboratory at the Institute for Forest Resources & Environment of Guizhou, Guizhou University. These fungi were subcultured on modified Melin Norkran’s (MMN) medium (Marx 1969) at 25°C in the dark. The MMN medium composition included: 25 mg/L NaCl, 250 mg/L (NH_4_)_2_HPO_4_, 500 mg/L KH_2_PO_4_, 5 mg/L FeCl_3_, 50 mg/L CaCl_2,_ 150 mg/L MgSO_4_·7H_2_O, 100 mg/L VB1, 10 g/L glucose, 1.00 g/L casamino acids, 5.00 g/L malt, and 10 g/L agar. *Fusariumoxysporum* is a type pathogenic fungus that caused root rot in trees, was obtained from the China General Microbiological Culture Collection Center and maintained on a medium containing glucose, peptone, yeast extract, and agar at 28°C. All fungi were cultured until suitable for inoculation.

*Pinusmassoniana* seeds were collected from the high-quality breeding base of *P.massoniana* in Maanshan Forest Farm, Duyun City, Guizhou Province, China. Seeds were surface sterilized following Feng et al. (2022) and grown in a climate chamber for 28 days. Wildtype *Arabidopsisthaliana* ecotype Columbia-1 (col1) seeds were provided by Prof. Fuhua Fan, and the T-DNA insertion mutant lines, including *yuc1*, *aux1*, *pin1*, *pin3*, *arf19*, and *tir1*, were purchased from the AraShare Technology Service Center (Table 1). *A.thaliana* seeds were sterilized with 75% alcohol and stratified at 4°C for 3 days before use.

**Table 1.** Information on auxin-related mutants in *A.thaliana*.

Mutant	Mutant type	Gene ID	Description	Pathway involved
*yuc1*	T-DNA insertion	*At4g32540*	auxin biosynthesis	auxin synthesis
*aux1*		*At2g38120*	auxin influx carrier	auxin polar transport and distribution
*pin1*		*At1g73590*	auxin efflux carrier	
*pin3*		*At1g70940*	auxin efflux carrier	
*arf19*		*At1g19220*	auxin response factor 19	auxin signaling and responses
*tir1*		*At3g62980*	transport inhibitor response 1	

### ﻿Experimental methods

#### ﻿Experiment 1: in vitro interaction between *P.massoniana* and *S.bovinus* via VOCs

Following the experimental procedure described by Feng et al. (2022), bicompartmented Petri dishes (13 × 13 cm) were prepared, with basal medium (DCR; composed of 400 mg/L NH_4_NO_3_, 556 mg/L Ca(NO_3_)_2_·4H_2_O, 370 mg/L MgSO_4_·7H_2_O, 85 mg/L CaCl_2_·2H_2_O, 170 mg/L KH_2_PO_4_, 6.2 mg/L H_3_BO_3_, 22.3 mg/L MnSO_4_·H_2_O, 8.6 mg/L ZnSO_4_·7H_2_O, 0.25 mg/L CuSO_4_·5H_2_O, 0.83 mg/L KI, 0.025 mg/L CoCl_2_·6H_2_O, 0.025 mg/L LiCl, 0.25 mg/L NaMoO_4_·2H_2_O, 27.8 mg/L FeSO_4_·7H_2_O, 37.3 mg/L EDTA−2Na, 1.0 mg/L VB1, 0.5 mg/L VB6, 0.5 mg/L nicotinic acid, 2.0 mg/L glycine, 200 mg/L myo−inositol, 10 g/L sucrose, and 10 g/L agar) (Gupta and Durzan 1985) in one compartment, and MMN medium in the other. The MMN medium was inoculated with a mycelial plug of either *S.bovinus*, *S.luteus*, *S.citrinum*, *P.fortinii*, *L.perlatum*, or *F.oxysporum*. A 28-day-old *P.massoniana* seedling was placed in the DCR medium compartment. Non-inoculated dishes served as controls (10 replicates per treatment). Dishes were incubated in a 25°C climate chamber with a 14-hour light and 10-hour dark photoperiod. Root growth was monitored weekly, and root length, branching, and biomass were measured after 28 days.

*Pinusmassoniana* exposed to *S.bovinus*VOCs in bicompartmented Petri dishes for 28 days were utilized for plant hormone assays. Three biological replicates of both the control (NM) and *S.bovinus* VOC treatment were collected as fresh tissue. Five seedlings from each treatment were combined to form one replicate. All samples were subsequently frozen in liquid nitrogen and stored at −80°C until the phytohormone assay was conducted. Plant hormones were quantified using ultra-performance liquid chromatography (UPLC) and tandem mass spectrometry. The liquid phase conditions were set according to the methodologies outlined by Cai et al. (2014), Niu et al. (2014), and Hua-Ming et al. (2018), and mass spectrometry conditions were based on the protocols of Pan et al. (2010), Simura et al. (2018), and Cui et al. (2015).

A “Y”-shaped PVC pipe system (Suppl. material 1: fig. S1a) was also used to study root growth in response to *S.bovinus*VOCs under similar conditions. Root growth was observed after 60 days.

**Figure 1.**
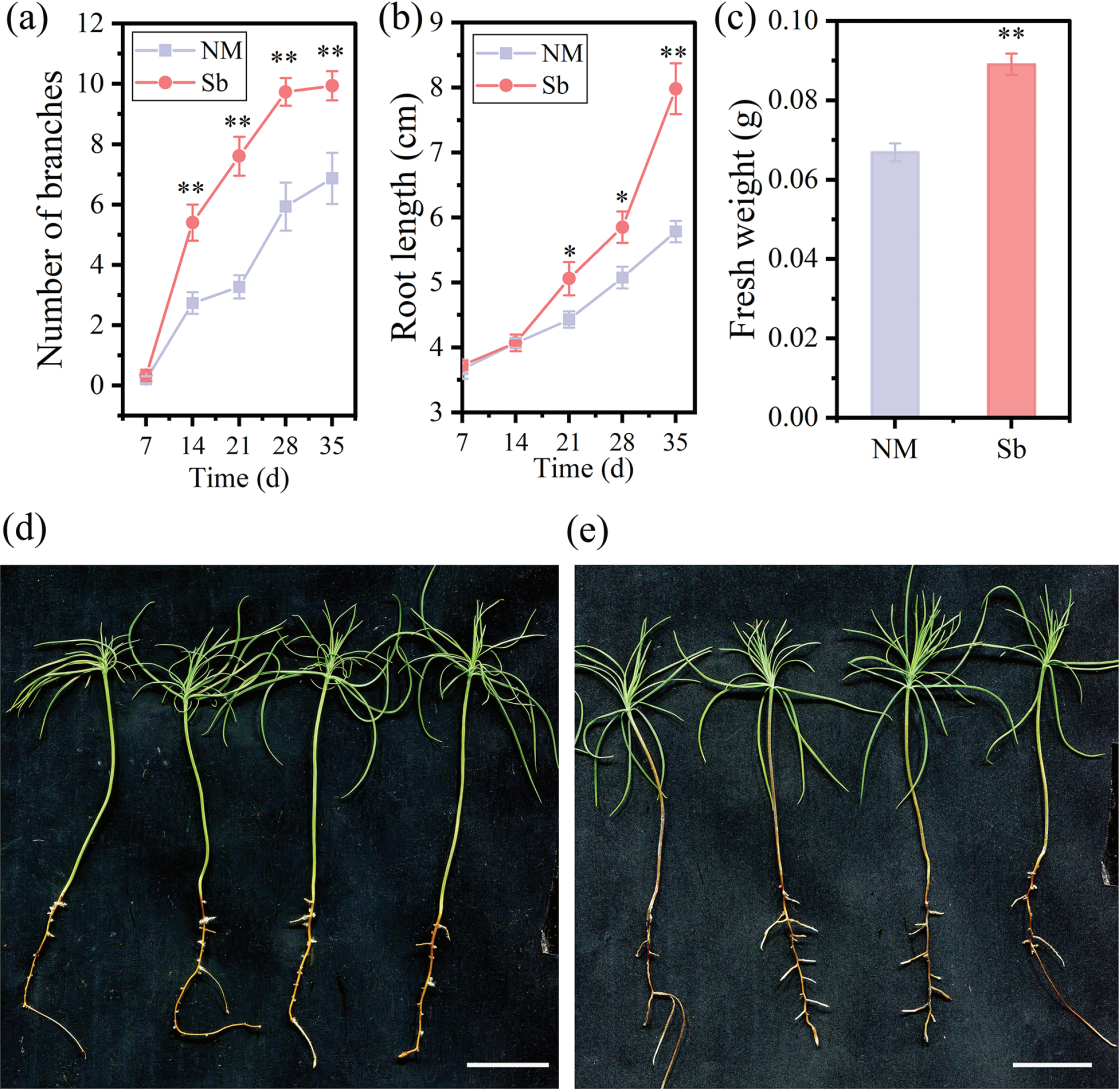
Effects of volatile organic compounds (VOCs) emitted by *Suillusbovinus* (Sb) on the growth of *Pinusmassoniana*. Seedlings grown in the absence of *S.bovinus*VOCs acted as controls (NM). **a** Number of branches, **b** root length, and **c** fresh weight of *P.massoniana*. n = 10. Data shown are mean values ± the standard error. * *P* < 0.05, ** *P* < 0.01. *P.massoniana* seedlings grown in the **d** absence and **e** presence of *S.bovinus*VOCs for 28 days. Scale bars: 2 cm.

#### ﻿Experiment 2: impact of SQTs emitted by *S.bovinus* on plant growth

##### Fungal VOC collection and identification

To investigate the volatiles signaling utilized by symbiotic fungi to enhance plant root growth, we compared the volatile profiles of four symbiotic fungi: *S.bovinus*, *S.luteus*, *S.citrinum*, and *P.fortinii*. MMN medium inoculated with a plug of each fungus was cultured in headspace vials at 25°C for 14 days (6 replicates). VOCs were collected using headspace-solid phase microextraction (SPME) and analyzed by gas chromatography-mass spectrometry (GC-MS). VOC identification was performed using selected ion monitoring. Fungal VOC identification and analysis were conducted by Metware Biotechnology Inc. (http://www.metware.cn; Wuhan, China).

##### Impacts of SQTs emitted by *S.bovinus* on plant growth

β-Cedrene (CAS:546–28–1; 95%; Shanghai Macklin Biochemical Co., Ltd, Shanghai, China) and α-humulene (CAS:6753-98-6; 93%; Shanghai Yuanye Bio-Technology Co., Ltd, Shanghai, China) were dissolved in anhydrous ethanol to prepare solutions with concentrations of 1, 10, 100 and 1000 ppb. According to the method of Ditengou et al. (2015), *P.massoniana* seedlings and wildtype *A.thaliana* seeds were exposed to various concentrations of β-cedrene or α-humulene in bicompartmented Petri dish. Fifty microlitres of β-cedrene or α-humulene solution was dropped on the filter paper (2 cm diameter), and controls received 50 μl of ethanol. Lovastatin (CAS:75330-75-5; 98%; Solarbio, Beijing, China), an inhibitor of fungal SQT production (Bach and Lichtenthaler 1982; Rodríguez-Concepción 2006), was also tested. The *P.massoniana* (28-day-old) seedlings or wildtype *A.thaliana* seeds were cultivated in one compartment of the bi-compartmented system and subjected to five different treatments, which were performed in the other compartment. (i) A 1-cm-diameter *S.bovinus* plug inoculated on MMN medium; (ii) Not inoculated with *S.bovinus* plug on MMN medium; (iii) A 1-cm-diameter *S.bovinus* plug inoculated on MMN medium containing lovastatin; (iv) A 1-cm-diameter *S.bovinus* plug inoculated on MMN medium containing lovastatin, and supplemented a filter paper with 50 μl of 1000 ppb (or 10 ppb for *A.thaliana* seeds) β-cedrene; (v) A 1-cm-diameter *S.bovinus* plug inoculated on MMN medium containing lovastatin, and supplemented a filter paper with 50 μl of 100 ppb (or 10 ppb for *A.thaliana* seeds) α-humulene solution. Plants of each treatment were grown under the same condition as in Experiment 1. Plant growth was monitored, and root length, branching, and biomass were measured. For *A.thaliana*, branch numbers were recorded after 20 days.

##### Role of the auxin pathway in *S.bovinus* VOC effects

Solutions of 1‐*N*‐naphthylphthalamic acid (NPA; CAS:132-66-1; Shanghai Macklin Biochemical Co., Ltd; an inhibitor of auxin transport) were prepared at final concentrations of 1, 3, and 5 μM. Additionally, solutions of yucasin (CAS:26028-65-9; Shanghai Macklin Biochemical Co., Ltd; an inhibitor of auxin synthesis) were prepared at final concentrations of 10, 50, and 100 μM. Different concentrations of NPA and yucasin were applied to *P.massoniana* seedlings and *A.thaliana* seeds exposed to *S.bovinus*VOCs or β-cedrene/α-humulene. Significantly, 50 μM yucasin limited *A.thaliana* seeds germination, so the concentration of yucasin solutions were adjusted to 1, 2, 5 or 10 μM. Plant growth parameters were recorded. The effects of *S.bovinus*VOCs, β-cedrene or α-humulene on *A.thaliana* IAA-associated mutants were also tested by a bicompartmented system.

#### ﻿Experiment 3: effects of exogenous SQTs on ECM formation

*Pinusmassoniana* seedlings were grown in a sterilized substrate (comprising peat, perlite, and vermiculite (3:1:1 by volume)) under controlled conditions. Four treatments were applied: inoculated with *S.bovinus*, inoculated with *S.bovinus* and supplemented with 1000 ppb β-cedrene, inoculated with *S.bovinus* and supplemented with α-humulene, and uninoculated. β-Cedrene and α-humulene were mixed with Hoagland solution and added once a week. Only Hoagland solution was added to the seedlings of the uninoculated and inoculated with *S.bovinus*. There were 30 pots of each treatment. After 90 days, mycorrhizal infection, seedling height, biomass, and root/shoot ratio were measured. Observe the ECM microstructure with reference to the method of Feng et al. (2022), and measure the thickness of mantle and the depth of Hartig net. Hartig net depth and mantle thickness were quantified using ImageJ software (http://rsbweb.nih.gov/ij/) by analyzing five independent root tips per treatment, with five measurements recorded per micrograph and then averaged.

### ﻿Statistical analysis

Data were analyzed using SPSS 25.0. Significant differences were determined by Student’s t-test or ANOVA with Duncan’s test (*P* < 0.05). Graphs were produced using Origin 2021. Venn diagrams of fungal VOCs were generated using Venny 2.1.0. (https://bioinfogp.cnb.csic.es/tools/venny/index.html).

## ﻿Results

### ﻿Effects of fungal VOCs on the growth of *P.massoniana*

**Figure 2.**
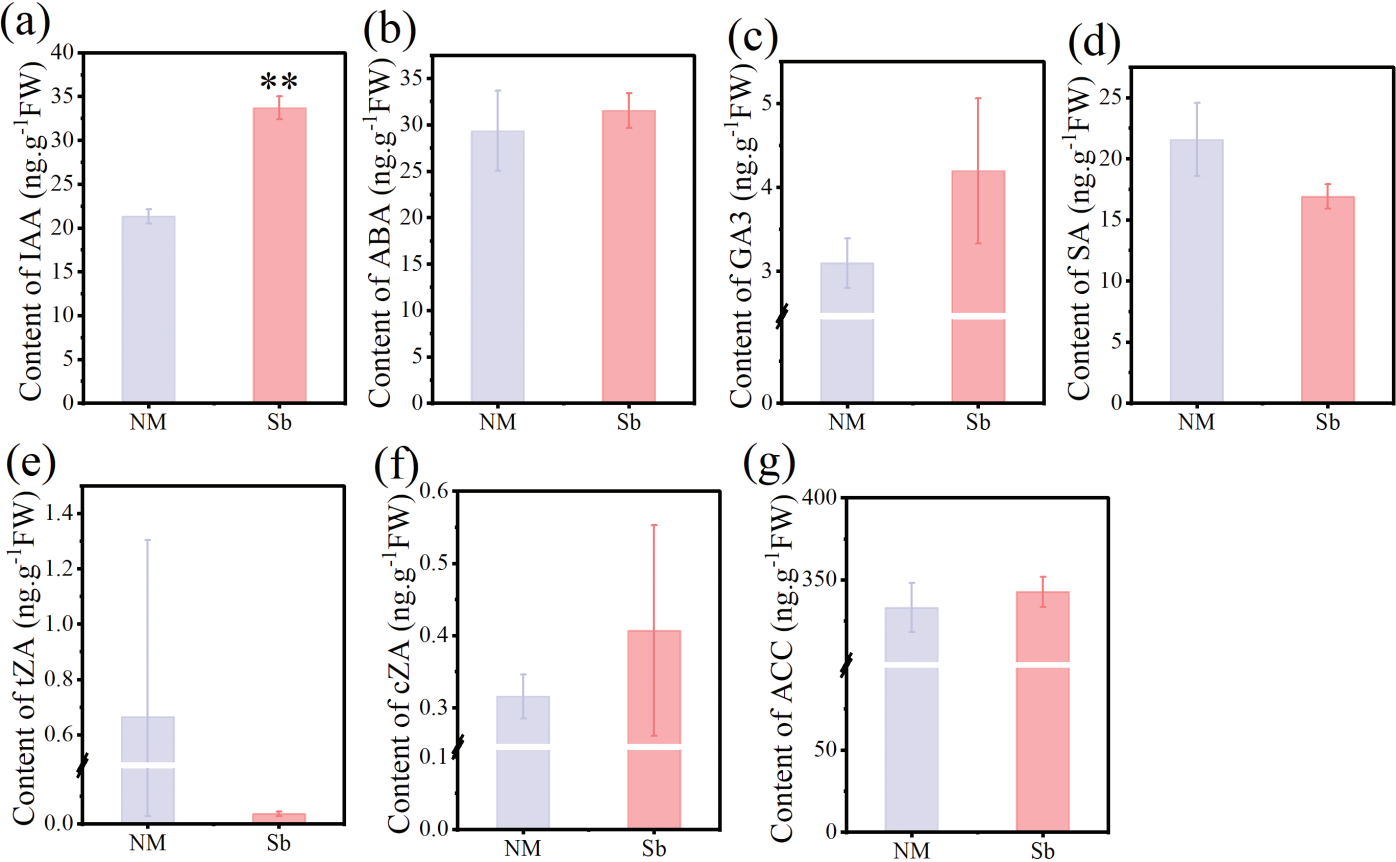
Effects of *Suillusbovinus* (Sb) volatile organic compounds (VOCs) on phytohormone accumulation in *Pinusmassoniana*. Seedlings grown in the absence of *S.bovinus*VOCs acted as controls (NM). Phytohormones: **a** indole-3-acetic acid (IAA); **b** abscisic acid (ABA); **c** gibberellic acid (GA3); **d** salicylic acid (SA); **e** trans-zeatin (tZA); **f** cis-zeatin (cZA); **g** 1-aminocyclopropane-1-carboxylate (ACC). Data shown are mean values ± the SE. *n* = 3. ** *P* < 0.01.

To investigate the impact of VOCs produced by ECM fungi on plant growth, we examined the effects of *S.bovinus*-emitted VOCs on root growth and plant hormone levels in *P.massoniana*. After 28 days of exposure to *S.bovinus*VOCs, the *P.massoniana* lateral root number, root length and biomass were 46.97% (Fig. 1a), 29.04% (Fig. 1b), and 35.48% greater (Fig. 1c), respectively, than those of *P.massoniana* grown in the absence of *S.bovinus*VOCs. IAA accumulation in *P.massoniana* exposed to *S.bovinus*VOCs was 58.11% greater than that of *P.massoniana* grown in the absence of *S.bovinus*VOCs but had no significant effect on the accumulation of other phytohormones (Fig. 2). In the “Y-tube” experiment, roots grew toward the pipe containing the *S.bovinus* culture, with minimal or no growth toward the uninoculated medium (Suppl. material 1: fig. S1b).

**Figure 3.**
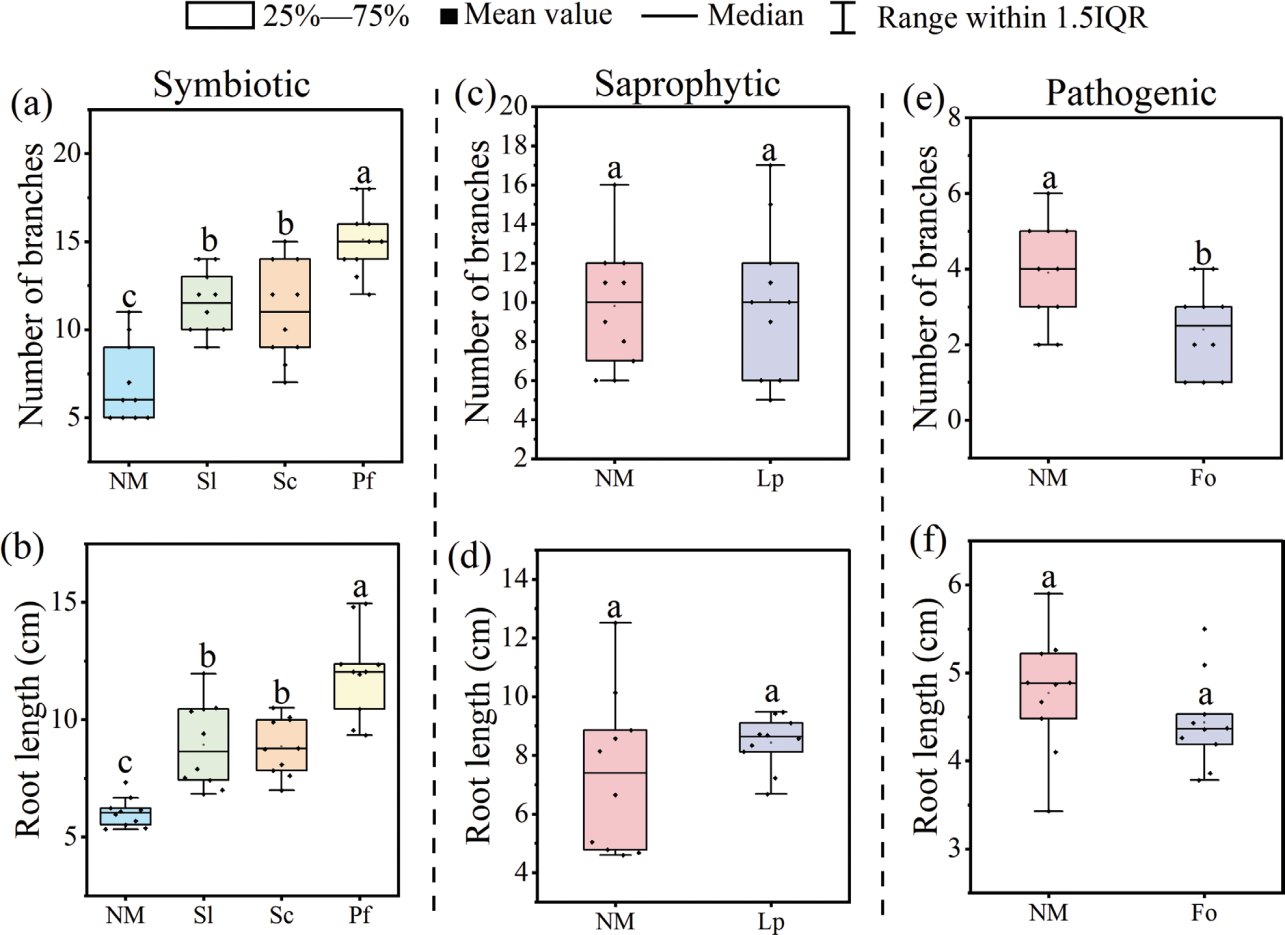
Effects of volatile organic compounds (VOCs) emitted by three different types of fungi on the root growth of *Pinusmassoniana*. **a–f** represent the number of root branches and root length of *Pinusmassoniana* exposed to VOCs emitted by symbiotic fungi (Sl, *Suillusluteus*; Sc, *Sclerodermacitrinum*; Pf, *Phialocephalafortinii*), a saprophytic fungus (Lp, *Lycoperdonperlatum*) and a pathogenic fungus (Fo, *Fusariumoxysporum*), respectively. Due to the fast growth rate of *F.oxysporum*, only 14 days of *P.massoniana* root growth data were collected, while the rest of the fungi collected 28 days of data. *n* = 10. NM, *P.massoniana* grown in the absence of fungal VOCs. Different letters above bars indicate significant differences between treatments (*P* < 0.05).

To assess the specificity of the effects of *S.bovinus*VOCs on plant growth, we examined the influence of VOCs emitted by other symbiotic, pathogenic, and saprophytic fungi on the root growth of *P.massoniana*. *P.massoniana* exposed to VOCs emitted by three other symbiotic fungi (i.e., *S.luteus*, *S.citrinum*, or *P.fortinii*) also showed significantly enhanced growth, including LRF and root elongation (Fig. 3a, b). However, the root growth of *P.massoniana* exposed to saprophytic fungi *L.perlatum*VOCs was not significantly different to that of the control (Fig. 3c, d). Conversely, the LRF of *P.massoniana* exposed to VOCs emitted by pathogenic fungi *F.oxysporum* was inhibited (Fig. 3e, f).

### ﻿VOCs profiles of symbiotic fungi

To identify the signaling compounds in VOCs that promote plant root growth, we compared the volatile profiles of four symbiotic fungi: *S.bovinus*, *S.luteus*, *S.citrinum*, and *P.fortinii*. The VOC spectrum emitted by four symbiotic fungi, including terpenoids, alcohols, esters, hydrocarbons, ketones, and heterocyclic compounds, etc (Suppl. material 1: table S1). Terpenoids were the most abundant, with SQTs being particularly prevalent. Among the four symbiotic fungi, the *S.bovinus* VOC profile had the largest proportion of terpenoids (63.83%) and the *P.fortinii* VOC profile had the smallest proportion of terpenoids (20.00%) (Suppl. material 1: fig. S2b). The VOCs produced by DSE *P.fortinii* promote *P.massoniana*LRF more effectively than those produced by ECM fungi; however, there was no significant difference in the VOC effects among the ECM fungi (Fig. 3a). Only twenty-six VOCs were shared by all three ECM fungi, nine of which were terpenoids, most of which were SQTs (7) (Table 2; Suppl. material 1: fig. S2a). β-Cedrene and α-humulene were among the shared SQTs produced by the three ECM fungi but were not present in the *P.fortinii* VOC profile. Previous researches described that these two SQTs analogues, such as β-caryophyllene (Minerdi et al. 2011; Yamagiwa et al. 2011) and cedrene (Li et al. 2022) can promote plant growth, therefore, it is speculated that the terpenoids β-cedrene and α-humulene play an important role in the presymbiotic stage between *P.massoniana* and *S.bovinus*.

**Table 2.** Twenty six shared volatile organic compounds (VOCs) emitted by *Suillusbovinus*, *Suillusluteus*, and *Sclerodermacitrinum*.

Formula	Compounds	Class	CAS
C5H8O	3-Pentyn-1-ol	Alcohol	10229-10-4
C7H8O2	Benzenemethanol, 4-hydroxy-	Alcohol	623-05-2
C10H8	Naphthalene	Aromatics	91-20-3
C10H12	Benzene, 1-methyl-3-(1-methylethenyl)-	Aromatics	1124-20-5
C10H12O3	Ethyl mandelate	Ester	774-40-3
C11H14O3	Benzoic acid, 4-ethoxy-, ethyl ester	Ester	23676-09-7
C6H6O2	Ethanone, 1-(2-furanyl)-	Heterocyclic compound	1192-62-7
C6H6O3	Isomaltol	Heterocyclic compound	3420-59-5
C13H28	Undecane, 2,4-dimethyl-	Hydrocarbons	17312-80-0
C11H24	Decane, 5-methyl-	Hydrocarbons	13151-35-4
C8H16	1-Octene	Hydrocarbons	111-66-0
C8H14	1,5-Heptadiene, 2-methyl-, (Z)-	Hydrocarbons	41044-64-8
C12H16O	3-Hexanone, 1-phenyl-	Ketone	29898-25-7
C13H20O	Butanone, 4-(2,6,6-trimethyl-1,3-cyclohexadien-1-yl)-	Ketone	20483-36-7
C10H18O3	2-Methylbutanoic anhydride	Others	1468-39-9
C2H6S3	Dimethyl trisulfide compounds	Sulfur compounds	3658-80-8
C6H14S2	Disulfide compounds, dipropyl	Sulfur compounds	629-19-6
C15H24	(1R,4R,4aS,8aR)-4,7-Dimethyl-1-(prop-1-en-2-yl)-1,2,3,4,4a,5,6,8a-octahydronaphthalene	Terpenoids	92692-39-2
C15H24	1,6,10-Dodecatriene, 7,11-dimethyl-3-methylene-	Terpenoids	77129-48-7
C15H24	Naphthalene, 1,2,3,5,6,8a-hexahydro-4,7-dimethyl-1-(1-methylethyl)-, (1S-cis)-	Terpenoids	483-76-1
C15H24	Spiro[4.5]dec-7-ene,1,8-dimethyl-4-(1-methylethenyl)-,[1S-(1α,4β,5α)]-	Terpenoids	24048-44-0
C15H24	1-Isopropyl-4,7-dimethyl-1,2,3,5,6,8 a-hexahydronaphthalene	Terpenoids	16729-01-4
C15H24	β-Cedrene	Terpenoids	546-28-1
C15H24	α-Humulene	Terpenoids	6753-98-6
C10H16	Cyclohexene, 1-methyl-4-(1-methylethylidene)-	Terpenoids	586-62-9
C13H20O	α-Ionone	Terpenoids	127-41-3

CAS: Chemical Abstracts Service Registry number.

### ﻿Effects of SQTs on plant growth

**Figure 4.**
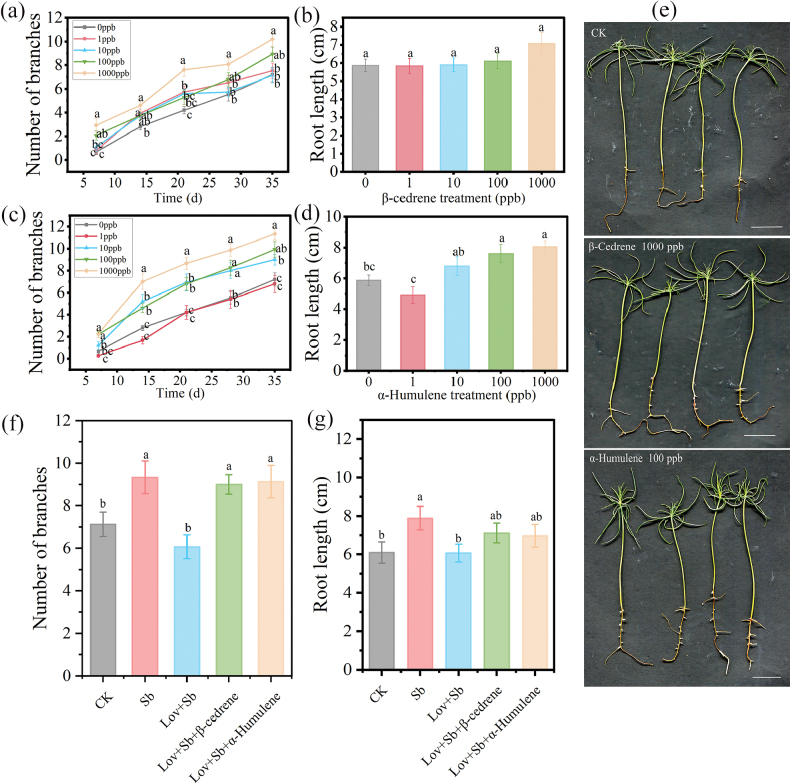
Effects of β-cedrene and α-humulene, sesquiterpenes (SQTs) emitted by *Suillusbovinus* (Sb), on the growth of *Pinusmassoniana*. Effects of different concentrations of β-cedrene on **a** root branching and **b** root length. Effects of different concentrations of α-humulene on **c** root branching and **d** root length. **e** Images of *P.massoniana* seedlings after 35 days of 1000 ppb β-cedrene or 100 ppb α-humulene treatment, CK, control seedlings; Scale bars: 2 cm. Effects of adding of β-cedrene or α-humulene to culture medium containing lovastatin (Lov) inhibits (which inhibits the synthesis of *S.bovinus* SQTs) on **f** root branching and **g** root length. *n* = 15. Error bars indicate ± the SE. Different letters between treatment after the same number of days of VOC exposure in (**a, c**), and different letters above the columns in (**b, d, f, g**) indicate significant differences between treatments (*P* < 0.05).

To investigate the growth-promoting effects of SQTs, we examined the impact of two SQTs, β-cedrene and α-humulene, common to the three ECM fungi, on root growth in the host *P.massoniana* and non-host *A.thaliana*. β-Cedrene and α-humulene both significantly promoted *P.massoniana* root development. After 35 days, the number of root branches of seedlings exposed to 1000 ppb β-cedrene was significantly greater than those exposed to 0, 1 or 10 ppb β-cedrene (Fig. 4a ,e). However, root length was not significantly affected by exposure to β-cedrene (Fig. 4b). By contrast, root development was promoted by 100 ppb α-humulene (Fig. 4e). Compared with seedlings in the control group (0 ppb), seedlings exposed to 100 ppb α-humulene exhibited a 37.92% increase in the number of root branches (Fig. 4c) and a 29.65% increase in root length (Fig. 4d). When the biosynthesis of SQTs by *S.bovinus* was blocked by lovastatin, there was no significant effect on *P.massoniana* root branching or root length. However, when 1000 ppb β-cedrene or 100 ppb α-humulene were also added to the fungal medium, *P.massoniana* root branching (Fig. 4f) and root length (Fig. 4g) were not significantly different to that of seedlings grown in the presence of *S.bovinus* without lovastatin.

**Figure 5.**
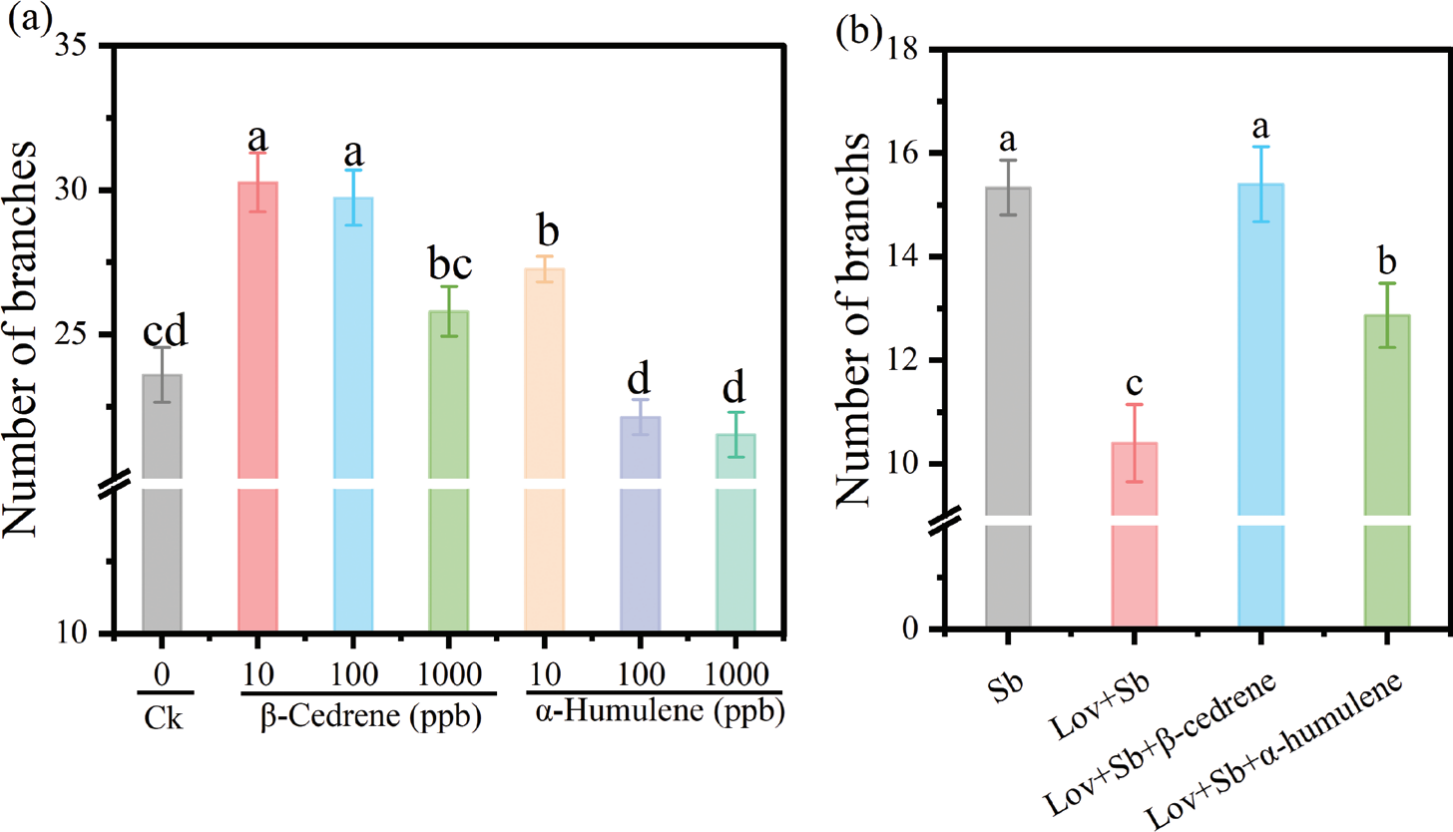
Effects of β-cedrene and α-humulene on *Arabidopsisthaliana* root growth. **a** Number of root branches produced by seedlings exposed to different concentrations of the sesquiterpenes (SQTs) β-cedrene or α-humulene; **b** effects of adding β-cedrene (10 ppb) or α-humulene (10 ppb) to culture medium containing lovastatin (Lov) (which inhibits the synthesis of *Suillusbovinus* (Sb) SQTs) on root branching, *n* = 15. Data shown are means ± the SE. Different letters above bars indicate significant differences between treatments (*P* < 0.05).

β-Cedrene and α-humulene also promote the LRF in the non-host *A.thaliana*. Concentrations of 10–100 ppb β-cedrene and 10 ppb α-humulene significantly increased the number of root branches (Fig. 5a). Furthermore, β-cedrene (10 ppb) and α-humulene (10 ppb) restored LRF in *A.thaliana* when lovastatin blocked SQT synthesis in *S.bovinus* (Lov + Sb + β-cedrene; Lov + Sb + α-humulene) (Fig. 5b).

### ﻿Role of auxin signaling pathway on VOC-induced effects

**Figure 6.**
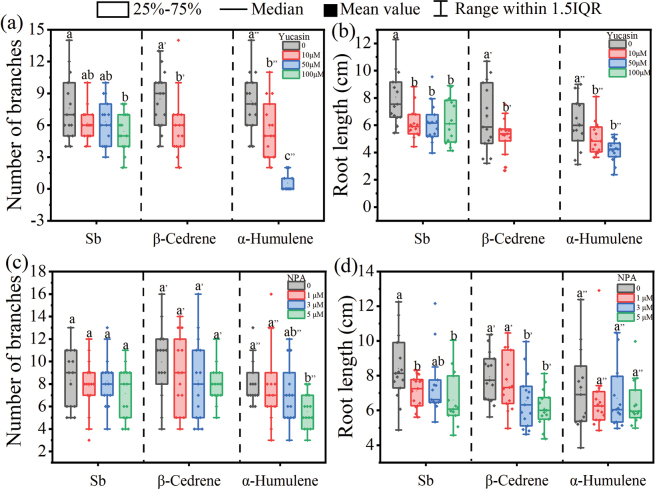
Effects of the auxin synthesis inhibitor yucasin and the auxin transport inhibitor 1-*N*-naphthylphthalamic acid (NPA) on *Pinusmassoniana* root development induced by *Suillusbovinus* volatile organic compounds (VOCs). Effects of adding different concentrations of the indole-3-acetic acid (IAA) synthesis inhibitor yucasin to medium inoculated with *S.bovinus*, or containing α-humulene or β-cedrene on **a** the number of *P.massoniana* root branches and **b** root length. The absence of some data for seedlings subjected to 50 M or 100 M yucasin is because *P.massoniana* wilted under these treatments. Effects of adding different concentrations of the IAA transport inhibitor NPA to medium inoculated with *S.bovinus* or containing α-humulene or β-cedrene on **c** the number of *P.massoniana* root branches and **d** root length. *n* = 15. Different letters above bars indicate significant differences between treatments (*P* < 0.05).

To investigate the involvement of the auxin signaling pathway in root growth induced by *S.bovinus*VOCs, we used an auxin inhibitor and *A.thaliana* auxin-related mutants to determine whether the root growth promotion effect was retained under different treatments. The promoting effect of *S.bovinus*, β-cedrene and α-humulene on *P.massoniana* root growth was repressed by the auxin synthesis inhibitor yucasin (Fig. 6a,b). At yucasin concentrations of 50 μM and 100 μM, *P.massoniana* lateral roots exposed to β-cedrene or α-humulene did not develop or even wilted (Fig. 6a). By contrast, the auxin transport inhibitor NPA had little effect on *P.massoniana* lateral root growth induced by *S.bovinus* or β-cedrene but weakened the promotion effect of α-humulene on root branching (Fig. 6c, d). At an NPA concentration of 5 μM, the number of *P.massoniana* lateral roots was significantly reduced by 30.83% (Fig. 6c) compared with those of control seedlings, which were not subject to NPA.

**Figure 7.**
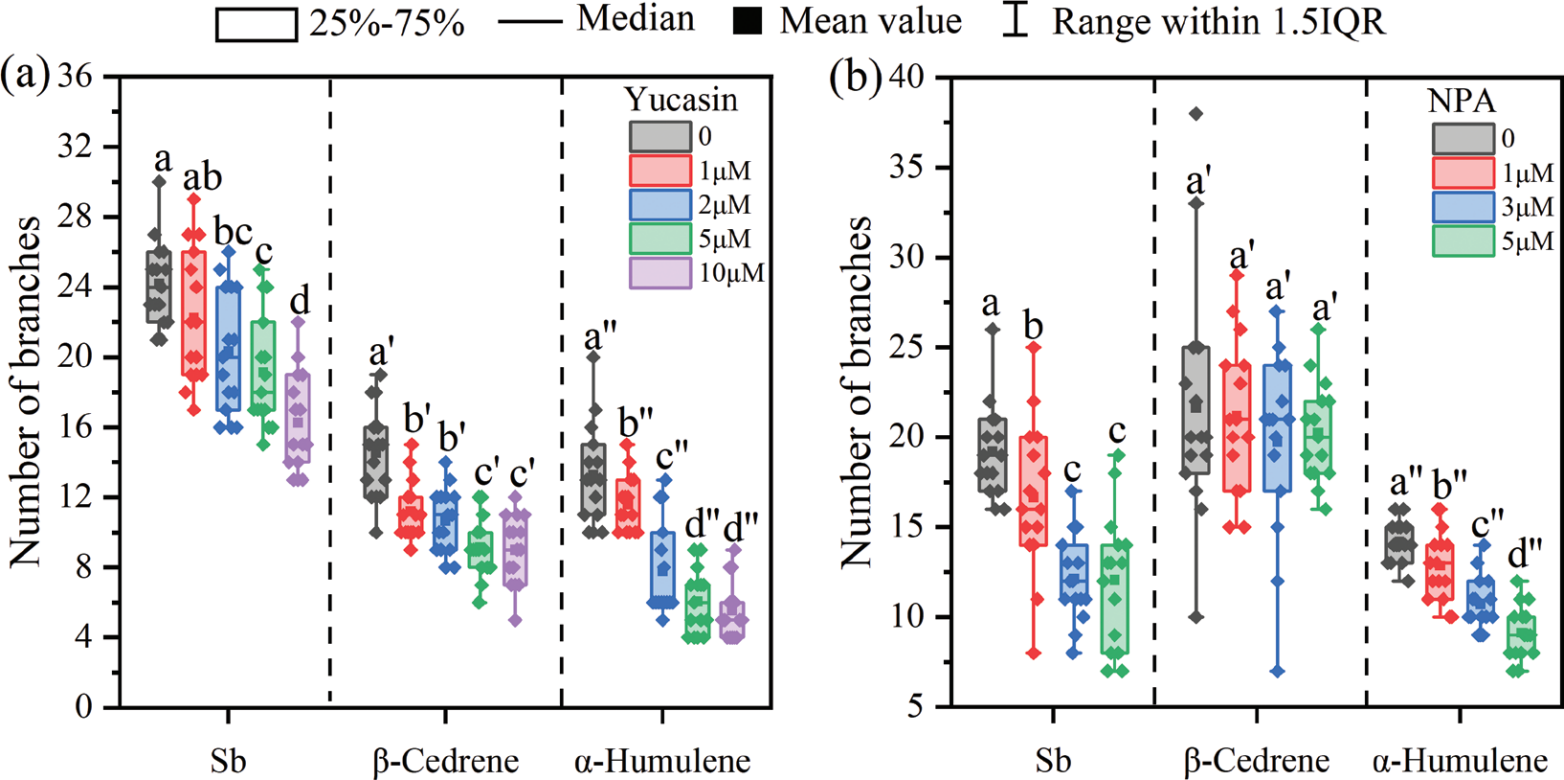
Effects of the auxin synthesis inhibitor yucasin and the auxin transport inhibitor 1‐*N*‐naphthylphthalamic acid (NPA) on *Arabidopsisthaliana* lateral root branch development induced by *Suillusbovinus* volatile organic compounds (VOCs). **a** Effects of adding different concentrations of yucasin to medium inoculated with *S.bovinus* or containing β-cedrene or α-humulene; **b** effects of adding different concentrations of NPA to medium inoculated with *S.bovinus* or containing β-cedrene or α-humulene. *n* = 15. Different letters above bars indicate significant differences between treatments (*P* < 0.05).

Similarly, both yucasin and NPA restricted the promoting of *S.bovinus*, β-cedrene, and α-humulene on the lateral root development of the nonhost *A.thaliana* (Fig. 7a, b). However, NPA treatment did not significant impact β-cedrene-induced LRF (Fig. 7b).

**Figure 8.**
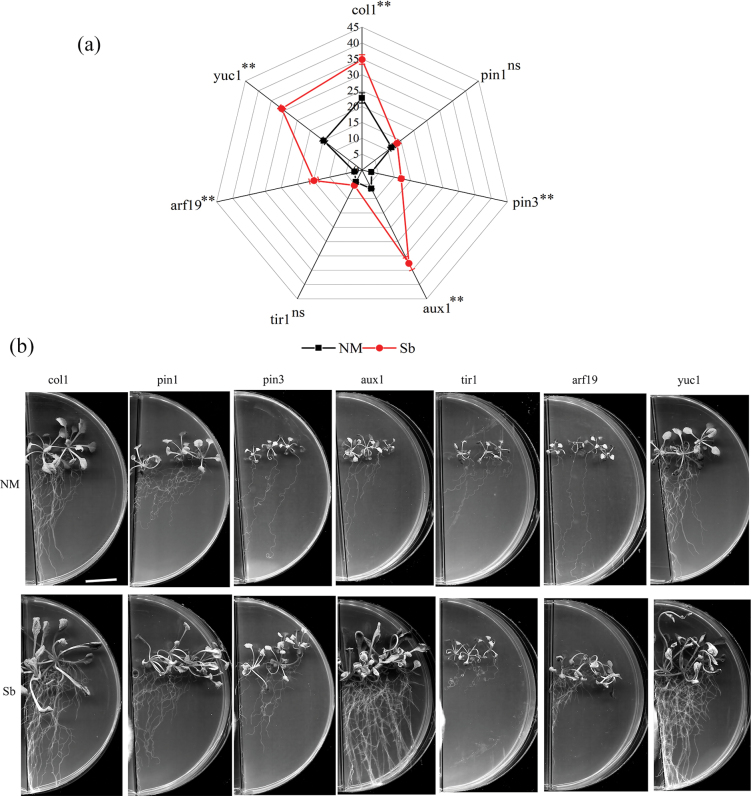
Effects of auxin-related gene function on *Suillusbovinus*-induced *Arabidopsisthaliana* root development. **a** Number of root branches in *A.thaliana* col1 wildtype and in the T-DNA insertion mutant lines *yuc1*, *aux1*, *pin1*, *pin3*, *arf19*, and *tir1* grown in the presence of *S.bovinus* (Sb) volatile organic compounds (VOCs) and in the absence of *S.bovinus*VOCs (NM) (*n* = 15). Error bars indicate ± the SE. *, *P* < 0.05; **, *P* < 0.01; ns, no significant difference between treatments. **b** Photographs showing the influence of *S.bovinus*VOCs on root architecture. Scale bar: 2 cm.

**Figure 9.**
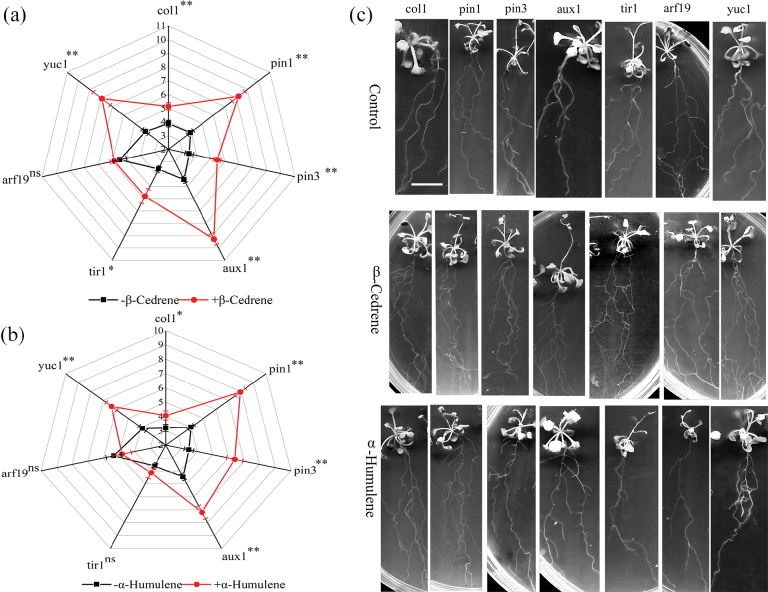
Effects of auxin-related gene function on β-cedrene- and α-humulene-induced *Arabidopsisthaliana* root development. Number of root branches in *A.thaliana* col1 wildtype and in the T-DNA insertion mutant lines *yuc1*, *aux1*, *pin1*, *pin*3, *arf19*, and *tir1* (**a**) grown in the presence of β-cedrene (shown in red) or in the absence of β-cedrene (shown in black), and (**b**) grown in the presence of α-humulene (shown in red) or in the absence of α-humulene (shown in black) (*n* = 15). Error bars indicate ± the SE. “*, *P* < 0.05; **, *P* < 0.01; “ns, no significant difference between treatments. **c** Photographs showing the influence of β-cedrene and α-humulene on root architecture. Scale bar: 2 cm.

Similar to the wildtype *A.thaliana* experiment, exposure to *S.bovinus*VOCs induced more root branching in most *A.thaliana* mutants compared with those that were not exposed to *S.bovinus*VOCs (NM treatment) (Fig. 8). Interestingly, exposure to *S.bovinus*VOCs did not increase the number of lateral roots in *pin1* and *tir1* lines (Fig. 8a) but did promote primary root elongation of the *pin1* line (Fig. 8b). The promotion of LRF by β-cedrene and α-humulene was not observed in the *arf19* line (Fig. 9a, c). Furthermore, α-humulene did not induce branching in the *tir1* line (Fig. 9b, c). Additionally, while *S.bovinus*VOCs seemed to stimulate shoot growth (Fig. 8b, data not collected), neither β-cedrene nor α-humulene had this effect (Fig. 9c).

### ﻿Effects of β-cedrene and α-humulene on the formation of an ECM symbiosis between *P.massoniana* and *S.bovinus*

**Figure 10.**
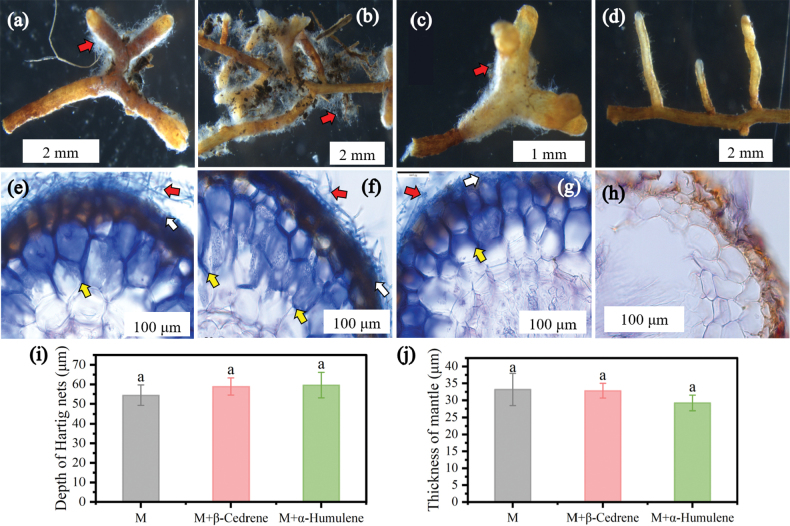
Effects of β-cedrene and α-humulene on the formation of an ectomycorrhizal (ECM) symbiosis between *Pinusmassoniana* and *Suillusbovinus*. Morphology of dichotomous root branches of seedlings subjected to: **a***S.bovinus* inoculation (M), **b** M+β-cedrene; **c** M+α-humulene treatment; or **d** no inoculation (NM). Cross-sections showing the microstructure of the *P.massoniana*–*S.bovinus*ECM association of seedlings subjected to: **e** M; **f** M+β-cedrene; **g** M+α-humulene; or **h** NM; the dye used was 0.03% trypan blue. **i** Depth of Hartig nets and **j** thickness of mantle under different treatments. Error bars indicate ± the SE, *n* = 5. Different letters above bars indicate significant differences between treatments. Red arrows indicate extraradical mycelium, white arrows indicate the mantle, and yellow arrows indicate Hartig nets.

To explore the role of SQTs in establishing ECM symbiosis, we observed the formation of ECM symbionts between *P.massoniana* and *S.bovinus* treated with β-cedrene and α-humulene. Overall, there was no significant difference in *P.massoniana* growth across NM, M, M+β-cedrene, and M+α-humulene treatments; however, the root development of seedlings inoculated with *S.bovinus* (M) was significantly greater than that of uninoculated seedlings (NM) (Suppl. material 1: table S2). Compared with the NM treatment, the M, M+β-cedrene and M+α-humulene treatments significantly increased the root/shoot ratio of *P.massoniana* by 47.06%, 47.06% and 41.18%, respectively (Suppl. material 1: table S2). Furthermore, the presence of β-cedrene and α-humulene increased the proportion of *P.massoniana* roots infected by *S.bovinus*, particularly β-cedrene (Suppl. material 1: fig. S3). Colonization of *P.massoniana* roots by *S.bovinus* led to the development of dichotomous branches mycorrhizae with well-developed mantle and Hartig net (Fig. 10). By contrast, under the NM treatment, there were no hyphae present between cells or on the root surface (Fig. 10d, h). The presence of β-cedrene or α-humulene did not significantly affect the depth of Hartig nets and the thickness of the mantle in the ECM symbiotic between *P.massoniana* and *S.bovinus* (Fig. 10i, j).

## ﻿Discussion

VOCs produced by microbes can diffuse through the atmosphere and soil, acting as crucial chemical signals in plant-microbe interactions. Previous studies have demonstrated that VOCs are involved in the signal exchange between plants and their fungal partners during the establishment of ECM symbioses (Ditengou et al. 2015; Garcia et al. 2015; Daguerre et al. 2016). In this study, we investigated the VOCs involved in the interaction between *P.massoniana* roots and *S.bovinus* during the presymbiotic stage and elucidated their mechanisms of action. Our findings indicate that the SQTs, β-cedrene and α-humulene, mediate the auxin pathway to regulate plant root growth and development during the presymbiotic stage of the *P.massoniana* and *S.bovinus*ECM association.

### ﻿Symbiotic fungal VOCs promote plant root growth

Traditionally, the beneficial functions of rhizosphere microbes in plant growth and development were thought to rely on direct microbe–root contact (Bais et al. 2004; Beauregard et al. 2013). However, VOCs released by microorganisms have been shown to enhance plant growth from a distance (Ditengou et al. 2015; Li et al. 2022; He et al. 2023; Wang et al. 2023). In this study, *S.bovinus* significantly promoted LRF and roots elongation in *P.massoniana* via VOCs, without direct root contact. Additionally, it induced root growth toward the source of the VOCs. LRF was considered to be a typical morphology in the early stage of mycorrhizal colonization because it was enhancing mycelium–root contact opportunities (Barker et al. 1998). These findings suggested that *S.bovinus*VOCs facilitates the communication and recognition by promoting *P.massoniana*LRF and directional root growth during the presymbiotic stage. Significantly, the effect of *S.bovinus*VOCs in promoting LRF may not be host-specific (Feng et al. 2022).

In addition to VOCs emitted by *S.bovinus*, we observed that VOCs emitted by other symbiotic fungi, including the ECM fungi *S.luteus* and *S.citrinum*, as well as the DSE *P.fortinii*, also stimulated *P.massoniana*LRF. Previous studies have similarly demonstrated that VOCs from other ECM fungi (Ditengou et al. 2015) and non-ECM fungi (Li et al. 2022; He et al. 2023; Liu et al. 2024) exhibit comparable functions. These findings suggest that plant root responses to VOCs from beneficial symbiotic fungi may not be specific to particular fungal species. Interestingly, pathogens also interact with plants via VOCs before physical contact (Venturi and Fuqua 2013). In our study, we observed that VOCs released by *F.oxysporum* inhibited the root growth of *P.massoniana*. In contrast, Moisan et al. (2019) and Hernández-Calderón et al. (2018) reported that VOCs emitted by pathogens can promoted plant growth, similar to the effects of VOCs from symbiotic fungi. Conversely, other studies have shown that VOCs released by pathogens may not affect plant growth (Tahir et al. 2017). Variations in plant responses to pathogen-emitted VOCs may result from differences in VOCs composition and concentration. Additionally, Peng et al. (2021) found that saprophytic fungi can act as mycorrhizal partners to promote host plant growth, suggesting that their VOCs may also play a similar role. However, in this study, VOCs emitted by the saprophytic fungus *L.perlatum* had no significant effect on *P.massoniana* growth. This lack of effect may be attributed to differences in fungal species, or it may suggest that saprophytic fungi *L.perlatum* necessitate direct contact to facilitate plant growth, which deserves further study. In this study, we propose that *P.massoniana*’s response to VOCs depends on the relationship between the fungi and the plant: VOCs from mutualistic fungi tend to promote root growth, those from pathogens inhibit growth, and those from saprophytic fungi have no effect.

### ﻿SQTs mimic *S.bovinus* VOC-induced effects on LRF promotion

Previous studies have shown that the ECM fungus *Cenococcumgeophilum*, which does not release SQTs, fails to induce LRF before physical contact, indicating the importance of SQTs in this process (Agger et al. 2009; Ditengou et al. 2015). In this study, the emission of SQTs by *S.bovinus* was crucial for promoting lateral roots’ emergence. This conclusion was further supported by experiments using lovastatin, an inhibitor of SQT biosynthesis, which significantly reduced the positive effect of *S.bovinus*VOCs on *P.massoniana*LRF, with similar effects observed in the non-host *A.thaliana*. These findings are consistent with previous reports (Ditengou et al. 2015; Li et al. 2022) and underscore that the effects of *S.bovinus*VOCs are not specific to host plants.

Furthermore, we identified two SQTs, β-cedrene and α-humulene, which were exclusively present in the VOC profiles of the three ECM fungi tested, indicating that these two SQTs may be specific to ECM fungi. These SQTs were sufficient to stimulate LRF in both the host *P.massoniana* and the non-host *A.thaliana* in the absence of *S.bovinus*, and even when SQT biosynthesis was inhibited by lovastatin. The roles of these two SQTs in plant–microbe interactions have not been previously demonstrated; however, their analogs (such as β-caryophyllene and cedrene) have been shown to induce plant growth (Minerdi et al. 2011; Yamagiwa et al. 2011; Li et al. 2022). Our study demonstrates their function as signaling compounds in the interaction between *P.massoniana* roots and *S.bovinus* at the presymbiotic stage, which is a novel finding.

Further analysis of their roles during the symbiotic formation of the *P.massoniana*–*S.bovinus* symbiosis revealed that β-cedrene and α-humulene do not significantly influence ECM symbiosis development, including the formation of the mantle and Hartig net. This is consistent with findings by Plett et al. (2024), who also observed that the addition of terpenes did not affect Hartig net development. Given that SQTs enhance the proportion of *P.massoniana* roots infected by *S.bovinus*, we hypothesize that VOCs signaling molecules may increase the likelihood of contact between roots and ECM fungal hyphae, thereby promoting favorable conditions for ECM formation. Meanwhile, other signaling molecules, such as phytohormones like ABA, as reported by Hill et al. (2022), may play crucial roles in initiating symbiosis. However, it remains unclear whether other VOC profiles contain signaling molecules besides β-cedrene and α-humulene that are involved in communication during the ECM presymbiotic stage. Future research should focus on large-scale screening for additional volatile signaling compounds and further analysis of their molecular mechanisms to determine their role in triggering LRF.

### ﻿Promotion effect of SQTs on root development relies on the auxin signaling pathway

Auxin, a crucial plant hormone, is widely recognized for its role in regulating LRF (Fukaki and Tasaka 2009). Previous studies have shown that microbial VOCs stimulate root branching by activating the host auxin signaling pathway (Felten et al. 2009; He et al. 2023; Liu et al. 2024). In this study, we demonstrated that *S.bovinus*VOCs significantly enhanced *P.massoniana* root branching and increased IAA accumulation in *P.massoniana*. These findings highlight the pivotal role of auxin in root development triggered by *S.bovinus*VOCs. Experiments using auxin biosynthesis and transport inhibitors further supported this conclusion. Yucasin, an auxin biosynthesis inhibitor, attenuated the effects of *S.bovinus* β-cedrene and α-humulene on LRF in both host and non-host plants, suggesting that *YUC* –mediated auxin biosynthesis is crucial in *S.bovinus*VOCs-induced LRF. This corroborates findings by Li et al. (2022), Zhang et al. (2022) and Wang et al. (2021). However, Ditengou et al. (2015) reported that SQTs released by *L.bicolor* could stimulate LRF independently of the auxin pathway. The discrepancy between these findings and those of other studies may stem from differences in the VOC profiles of different fungal species. Moreover, studies have shown that disrupting auxin polar transport alters auxin accumulation in roots and affects root growth (Zamioudis et al. 2013; Li et al. 2022; He et al. 2023). For instance, the ECM host *Populus* and non-host *A.thaliana* exposed to *L.bicolor*VOCs showed restricted LRF after NPA treatment (Felten et al. 2009), and a recently reported study had similar results (Qin et al. 2024). In our study, only the non-host *A.thaliana* was affected by NPA treatment, whereas *S.bovinus*VOCs continued to promote LRF in the host plant *P.massoniana*. This suggests that *S.bovinus*VOCs may contain auxin-independent signaling molecules that stimulate *P.massoniana*LRF. Importantly, *S.bovinus* VOC effects are not host specific, and the underlying mechanism may vary, potentially operating differently in different plant species. Furthermore, we observed that α-humulene induced LRF in both *P.massoniana* and *A.thaliana* via auxin polar transport, whereas β-cedrene did not, indicating that different VOCs enhance LRF through distinct mechanisms.

Interestingly, *S.bovinus*VOCs, β-cedrene and α-humulene, promoted LRF in *A.thaliana yuc1* mutants, contrasting with the yucasin results, which inhibits auxin biosynthesis. Since 11 members of the *YUCCA* gene family have been detected in *A.thaliana* (Mashiguchi et al. 2011), other *YUCCA* genes may be involved in this process. The AUX1/LAX and PIN protein families have distinct roles in auxin influx and efflux, respectively (Pacifici et al. 2015). The effect of *S.bovinus*VOCs on *A.thaliana* lateral root growth was abolished in *pin1* mutants but remained unchanged in *aux1* mutants, indicating that PIN1 is involved in the development of lateral roots stimulated by *S.bovinus*VOCs. This further supports our hypothesis that *S.bovinus*VOCs stimulate *A.thaliana*LRF through auxin polar transport, requiring a functional auxin efflux system, as also suggested by previous studies (Sun et al. 2020; He et al. 2023). Treatment with NPA attenuated the ability of α-humulene to stimulate LRF in both *P.massoniana* and non-host *A.thaliana*, whereas β-cedrene was unaffected. Interestingly, experiments with *A.thaliana* mutants indicated that the promotion effects of α-humulene may not rely on auxin polar transport, given that it induced LRF in *aux1*, *pin1*, and *pin3* mutants. These results contradict the outcomes of the NPA inhibitor trail, suggesting that α-humulene might stimulate LRF through other auxin influx and efflux carriers, which warrants further investigation.

Auxin signal transduction is crucial for regulating plant LRF in response to microbial VOCs (Tyagi et al. 2018). Studies by Li et al. (2022) and He et al. (2023) suggest that microbial VOCs promote LRF through complete auxin signal transduction, a finding supported by our study. In plants, the primary auxin signaling pathway involves the auxin receptor TIR1/AFB, AUX/IAA signal response factors, and ARF transcription factors (Hayashi 2012). While *S.bovinus*VOCs continued to promote root branching in *A.thaliana arf19* mutants, neither β-cedrene nor α-humulene alone could induce LRF in these mutants, indicating that the regulatory mechanism of *S.bovinus*VOCs on root branching likely involves multiple signaling molecules rather than a single pathway.

Additionally, *S.bovinus*VOCs appeared to stimulate shoot growth in *A.thaliana*. This could be due to enhanced lateral root formation, which improves nutrient absorption and promotes shoot growth (Zhang et al. 2022), or the VOCs may contain unidentified plant growth regulators or signaling molecules, as neither β-cedrene nor α-humulene had this effect. Further research is needed to explore these hypotheses.

## ﻿Conclusion

**Figure 11.**
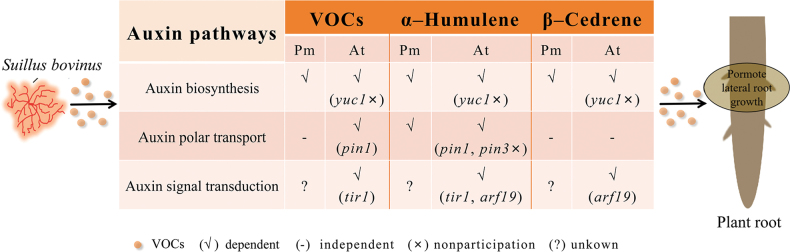
Schematic diagram illustrates the dependence of *Suillusbovinus* volatile organic compounds (VOCs) induced lateral root formation on auxin signaling pathways. Orange dots in the figure represent VOCs released by *S.bovinus*. In the table, a “√” indicates that the VOCs, α-humulene or β-cedrene, rely on the specific auxin pathway to stimulate plant root growth. A “-” means the induction is independent of that pathway, while a “?” denotes uncertainty about the pathway’s involvement. Genes listed in parentheses indicate their role in the process by which *S.bovinus*VOCs promote root growth via the corresponding pathway. A gene marked with an “×” in parentheses signifies its non-participation in this process. Pm: *Pinusmassoniana*; At: *Arabidopsisthaliana*.

Our study confirmed that *S.bovinus*VOCs can influence plant root architecture without direct contact with the plant, independent of host specificity, with SQTs identified as the primary VOC signals involved in this process. Specifically, α-humulene and β-cedrene, two specific SQTs, were identified as signaling molecules that modulate different auxin pathways to enhance host plant *P.massoniana* and nonhost *A.thaliana* root branching (Fig. 11). However, these findings alone do not fully explain how *S.bovinus*VOCs regulate LRF, necessitating further investigation. In addition, α-humulene and β-cedrene do not affect the ECM symbiotic relationship between *P.massoniana* and *S.bovinus*. In conclusion, our study underscores the role of SQTs in recognizing signals during the ECM presymbiotic stage and in regulating plant growth.

## ﻿Additional information

### Adherence to national and international regulations

All the fungal strains used in this study have been legally obtained, respecting the Convention on Biological Diversity (Rio Convention).

### Author ORCIDs

Wanyan Feng https://orcid.org/0000-0001-5645-6742

Xueguang Sun https://orcid.org/0000-0002-7895-6702

Guiyun Yuan https://orcid.org/0009-0005-5345-4238

Guijie Ding https://orcid.org/0009-0008-5760-0425

### Data availability

The original data presented in this study are included in the article/Suppl. material 1. Further inquiries can be directed to the corresponding author.

## Supplementary materials

Supplementary material 1 Supplementary tables and figures Data type docx Explanation note **table S1**: Volatile organic compounds (VOCs) of symbiotic fungi. **table S2**: Growth of *Pinusmassoniana* under different treatments. **fig. S1**: Volatile organic compound (VOC) localization experiment. **fig. S2**: Analysis of fungal volatile organic compounds (VOCs) produced by *Suillusbovinus* (Sb), *Suillusluteus* (Sl), *Sclerodermacitrinum*, and *Phialocephalafortinii*. **fig. S3**: Proportion of *Pinusmassoniana* seedling roots infected by Suillusbovinus under different treatments. NM, uninoculated seedlings (control); M, seedlings inoculated with *S.bovinus*. https://binary.pensoft.net/file/1289201This dataset is made available under the Open Database License (http://opendatacommons.org/licenses/odbl/1.0/). The Open Database License (ODbL) is a license agreement intended to allow users to freely share, modify, and use this Dataset while maintaining this same freedom for others, provided that the original source and author(s) are credited.Wanyan Feng, Xueguang Sun, Guiyun Yuan, Guijie Ding
