# Supplementary material for: ﻿Suillusbovinus sesquiterpenes stimulate root growth and ramification of host and non-host plants by coordinating plant auxin signaling pathways
Source: IMA Fungus. 2025 Mar 24;16:e142356. doi: 10.3897/imafungus.16.142356 (PMC11959287; doi:10.3897/imafungus.16.142356)
Supplement: Supplementary material 1 — Supplementary tables and figures [file imafungus-16-e142356-s001.docx]

***Suillus bovinus*** **sesquiterpenes stimulate root growth and ramification** **of host and nonhost plants by coordinating plant auxin signaling pathways**

Wanyan Feng^a,b,c^, Guiyun Yuan^a,b,c^, Xueguang Sun^a,b,c^ , Guijie Ding^a,b,c^

| VOCs | *Suillus bovinus* | *Suillus luteus* | *Scleroderma citrinum* | *Phialocephala fortinii* |
| --- | --- | --- | --- | --- |
| Terpenoids | 47 | 58 | 195 | 30 |
| Alcohols | 19 | 27 | 50 | 16 |
| Esters | 28 | 45 | 120 | 26 |
| Hydrocarbons | 22 | 39 | 47 | 20 |
| Ketones | 16 | 33 | 56 | 17 |
| Acids | 7 | 8 | 24 | 7 |
| Sulfur compounds | 8 | 11 | 15 | 6 |
| Heterocyclic compounds | 42 | 64 | 111 | 39 |
| Aromatics | 27 | 28 | 53 | 25 |
| Aldehydes | 12 | 21 | 33 | 12 |
| Nitrogen compounds | 3 | 4 | 7 | 2 |
| Phenols | 2 | 5 | 12 | 1 |
| Amines | ND | 3 | 10 | 1 |
| Ethers | ND | 1 | 1 | 1 |
| Halogenated hydrocarbons | ND | ND | 2 | ND |
| Others | 3 | 3 | 3 | 1 |

**Table S1** Volatile organic compounds (VOCs) of symbiotic fungi

ND, not detected.

**Table S2** Growth of *Pinus massoniana* under different treatments

| Treatments | Seedling height (cm) | Ground diameter (mm) | Shoot dry weight (g) | Root dry weight (g) | Total dry weight (g) | Root/shoot ratio |
| --- | --- | --- | --- | --- | --- | --- |
| NM | 16.34±0.53a | 1.98±0.17a | 0.81±0.11a | 0.13±0.01b | 0.94±0.12a | 0.17±0.01c |
| M | 17.02±0.52a | 2.26±0.12a | 0.85±0.07a | 0.21±0.02a | 1.06±0.07a | 0.25±0.02ab |
| M+β-cedrene | 16.64±0.37a | 1.94±0.08a | 0.68±0.07a | 0.17±0.02ab | 0.85±0.08a | 0.25±0.04a |
| M+α-humulene | 16.44±0.36a | 2.12±0.06a | 0.71±0.06a | 0.17±0.01ab | 0.88±0.09a | 0.24±0.01ab |

M, seedlings inoculated with *Suillus bovinus*; NM, uninoculated seedlings.

Data shown are mean values ± the SE. Different lowercase letters within a column indicate a significant difference between treatments (*P* < 0.05).

**
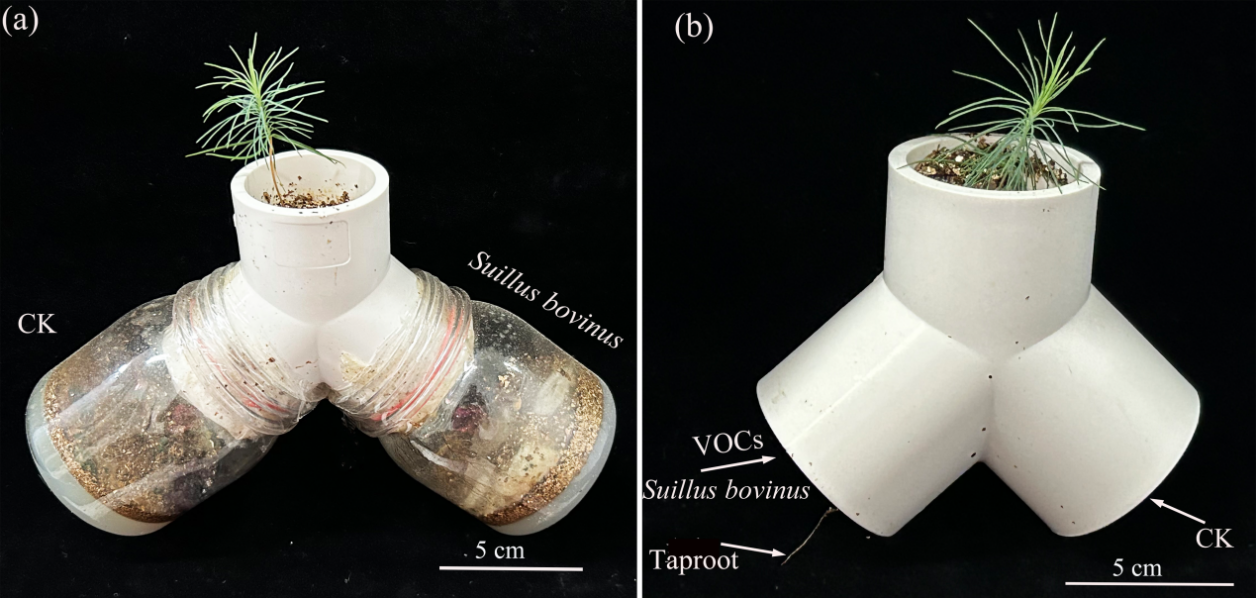
**

**Fig. S1** Volatile organic compound (VOC) localization experiment. (a) Schematic showing the assembly of the Y-shaped tube and culture bottles. In the test device, the diameter of PVC pipe is 40 mm, the diameter tissue culture bottle is 50 mm and the height is 90 mm. The middle part of the “Y” pipe was filled with a mixture of peat soil, vermiculite, and perlite (3:1:1 by volume). The ends of the “Y” pipe were connected to tissue culture bottles containing the nutrient soil mixture described above and MMN liquid medium. One bottle was inoculated with three *S. bovinus* plug, the other bottle was not inoculated and acted as the control. (b) Morphology of a *Pinus massoniana* seedling after two months of growth in a Y-shaped tube.

**
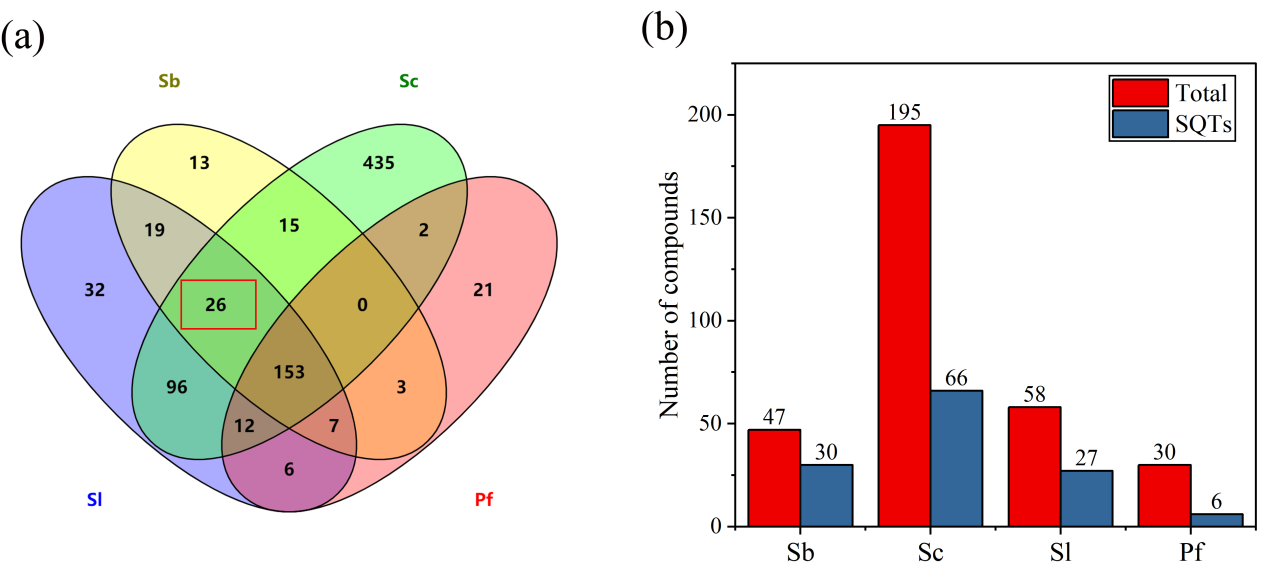
**

**Fig. S2** Analysis of fungal volatile organic compounds (VOCs) produced by *Suillus bovinus* (Sb), *Suillus luteus* (Sl), *Scleroderma citrinum*, and *Phialocephala fortinii*. (a) Venn diagram of the number of VOCs and (b) sesquiterpenes (SQTs) produced by each of the four fungi.

**
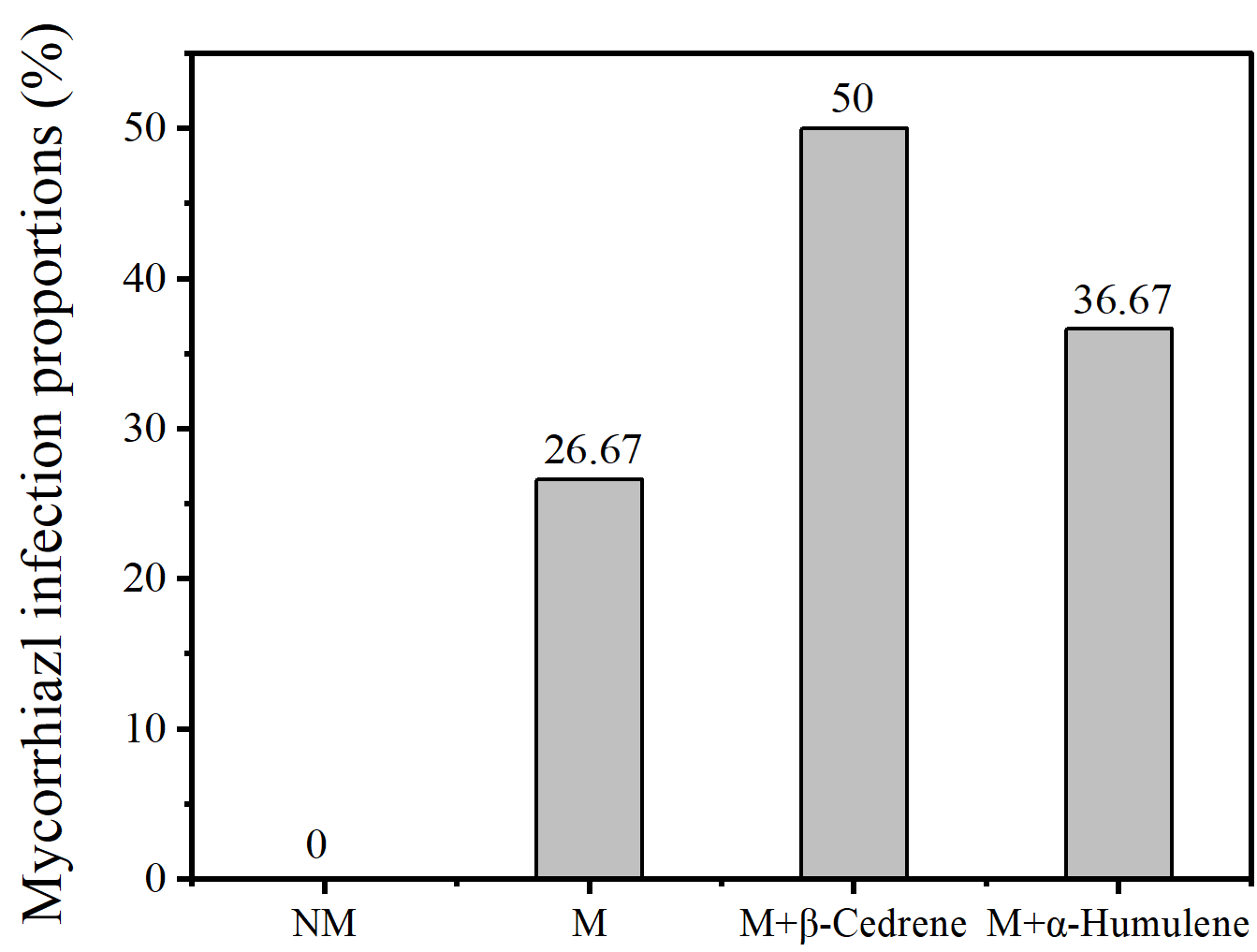
**

**Fig. S3** Proportion of *Pinus massoniana* seedling roots infected by *Suillus bovinus* under different treatments. NM, uninoculated seedlings (control); M, seedlings inoculated with *S. bovinus*.
